# Meconium peritonitis due to fetal appendiceal perforation: two case reports and a brief review of the literature

**DOI:** 10.1186/s12887-018-1133-8

**Published:** 2018-05-11

**Authors:** Yi Wang, Yeming Wu, Wenbin Guan, Wenbo Yan, Yuhua Li, Jin Fang, Jun Wang

**Affiliations:** 1 Department of Pediatric Surgery, Xinhua Hospital Affiliated to Shanghai Jiaotong University School of Medicine, No 1665, Kongjiang Road, Shanghai, 200092 People’s Republic of China; 2 Department of Pathology, Xinhua Hospital Affiliated to Shanghai Jiaotong University School of Medicine, Shanghai, 200092 China; 3 Department of Radiology, Xinhua Hospital Affiliated to Shanghai Jiaotong University School of Medicine, Shanghai, 200092 China

**Keywords:** Meconium peritonitis, Appendicitis, Intestinal duplication, Fetus, Surgery

## Abstract

**Background:**

Meconium peritonitis is an infrequent congenital disease usually caused by perforation of the fetal digestive tract. Meconium peritonitis resulting from intrauterine appendiceal perforation has been rarely reported and is often overlooked during pregnancy. We herein report two cases of fetal appendiceal perforation.

**Case presentation:**

Two neonates were found to have intestinal distension and gradually increasing ascites antenatally. After birth, diagnostic abdominal punctures revealed meconium peritonitis. Urgent surgery showed both neonates had developed gangrenous appendicitis in utero. Pathological examination supported the diagnosis of fetal appendiceal perforation in both neonates, and one also had deformity of cecal duplication. In the present report, we also review the presentation, diagnosis, pathology, management, and recent literature of fetal appendiceal perforation.

**Conclusion:**

Meconium peritonitis due to fetal appendiceal perforation is extremely rare, and preoperative diagnosis is almost impossible. However, clinicians should be aware of abnormal gastrointestinal manifestations in the fetus during the antenatal examination. For neonates with severe meconium peritonitis, an early operation with careful intraoperative exploration is necessary.

## Background

Meconium peritonitis (MP) is a sterile chemical peritonitis that is caused by intrauterine bowel perforation and has low morbidity (1/30,000). Although the mortality rate was historically high (70–80%), the survival rate for neonates with MP has increased to > 80% because of improvements in fetal diagnostic techniques and proper management, including surgical procedures [1, 2]. Local ischemia and bowel obstruction caused by complications such as enteric internal hernia or intestinal atresia can lead to intestinal walled-off necrosis [1, 2]. The distal ileum is the most common site of perforation, while perforation of the appendix is very rare in the fetal period; only a few cases have been reported to date.

We herein describe two newborns diagnosed with MP due to fetal appendiceal perforation, and we share our experience with special reference to the clinical presentation, evaluation (particularly with respect to the preoperative diagnosis and pathological diagnosis), and surgical treatment of this condition.

## Case presentation

### Case 1

**Fig. 1:**
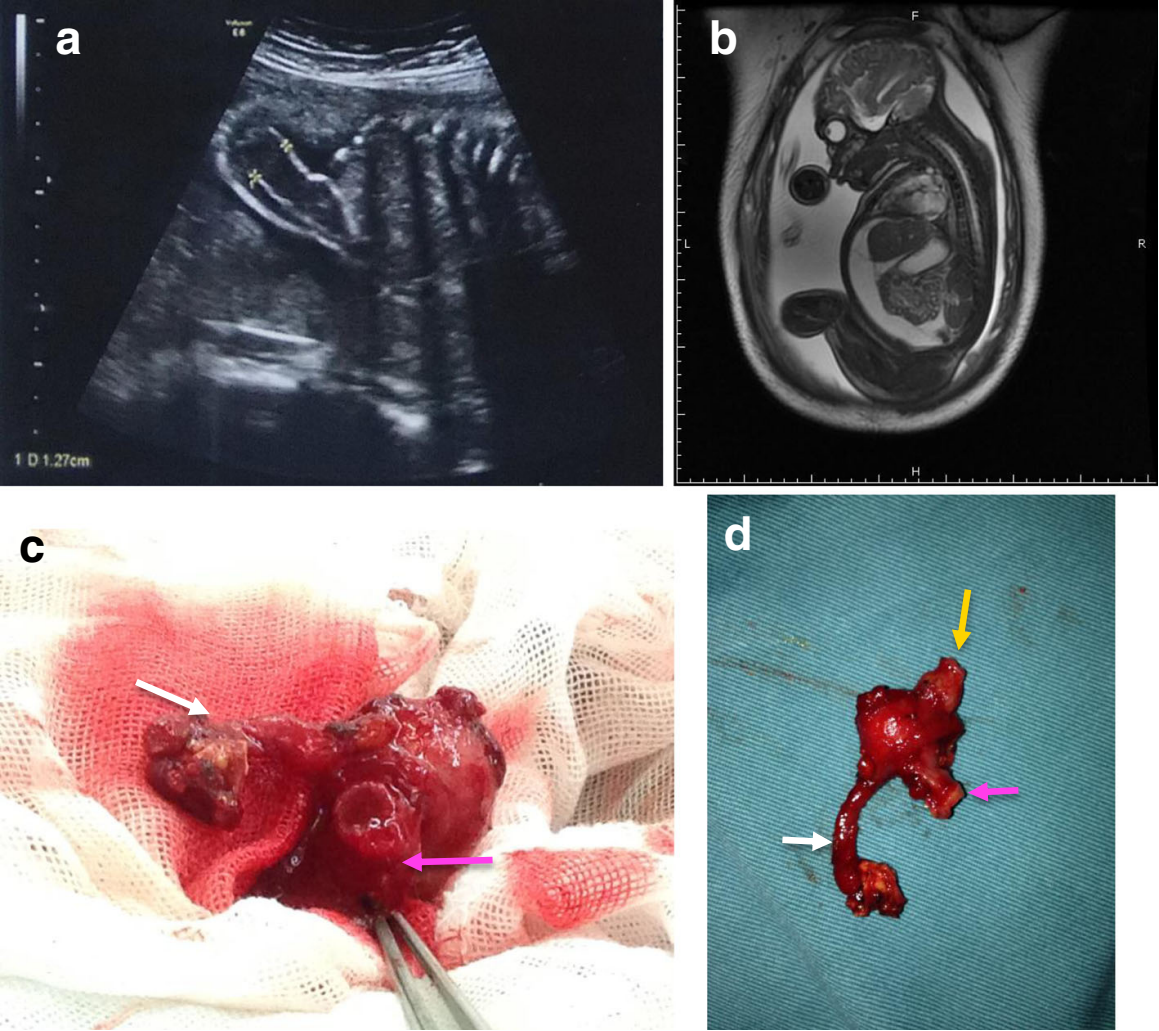
Imaging examination in fetal period and intraoperative finding for Case 1. **a** Prenatal US examination in 26-week gestation revealed the fetal intestine was dilated obviously as the inner diameter to 12.7 mm. **b** Sagittal MRI scanning in 34-week gestation showed the ascites had been increasing constantly with 32 mm in depth. **c**, **d** Gangrenous appendicitis with perforation was near the tip as well as a perforated lesion at the terminal ileum. We performed a limited ileocaecal resection with ileo-ascending colon anastomosis (white arrow: the gangrenous appendix, pink arrow: terminal ileum, yellow arrow: ascending colon)

A 3490-g boy was born by cesarean section at 36 5/7 weeks of gestation to a 26-year-old gravida 1, para 1. The Apgar scores at 5 and 10 min were 9 and 10, respectively, with normal oxygen saturation. Prenatal ultrasound (US) examination showed local intestinal expansion and mild polyhydramnios at 26 weeks of gestation (Fig. 1a). Magnetic resonance imaging (MRI) showed ascites in the fetal abdomen, and the ascites had been increasing in the metaphase and terminal period of gestation (Fig. 1b). Because a congenital digestive malformation was suspected based on the above-mentioned findings, the neonate was fasted and admitted to the intensive care unit from birth.

Physical examination of the neonate revealed normal development and a normal response to external stimulation. His abdomen was slightly distended without palpation of a mass; however, an asymmetric feeling of guarding was noted during abdominal palpation. Laboratory studies showed that the white blood cell count and C-reactive protein (CRP) concentration were normal. A plain abdominal radiograph and US examination revealed intestinal obstruction with ascites. The patient was then admitted to the hospital. Diagnostic abdominocentesis was performed, and yellow turbid liquid was extracted. Thus, the primary diagnosis of MP was made, and an urgent exploratory laparotomy was performed.

At surgery, about 300 ml of turbid ascites was aspirated, and all of the abdominal organs were encased within a huge cystic pseudomembranous structure. Dense adhesions were present around the gut with scattered calcifications. After releasing the adhesions, we found gangrenous appendicitis with perforation near the tip as well as a perforated lesion at the terminal ileum. A limited ileocecal resection was then performed with ileo–ascending colon anastomosis (Fig. 1c, d).

**Fig. 2:**
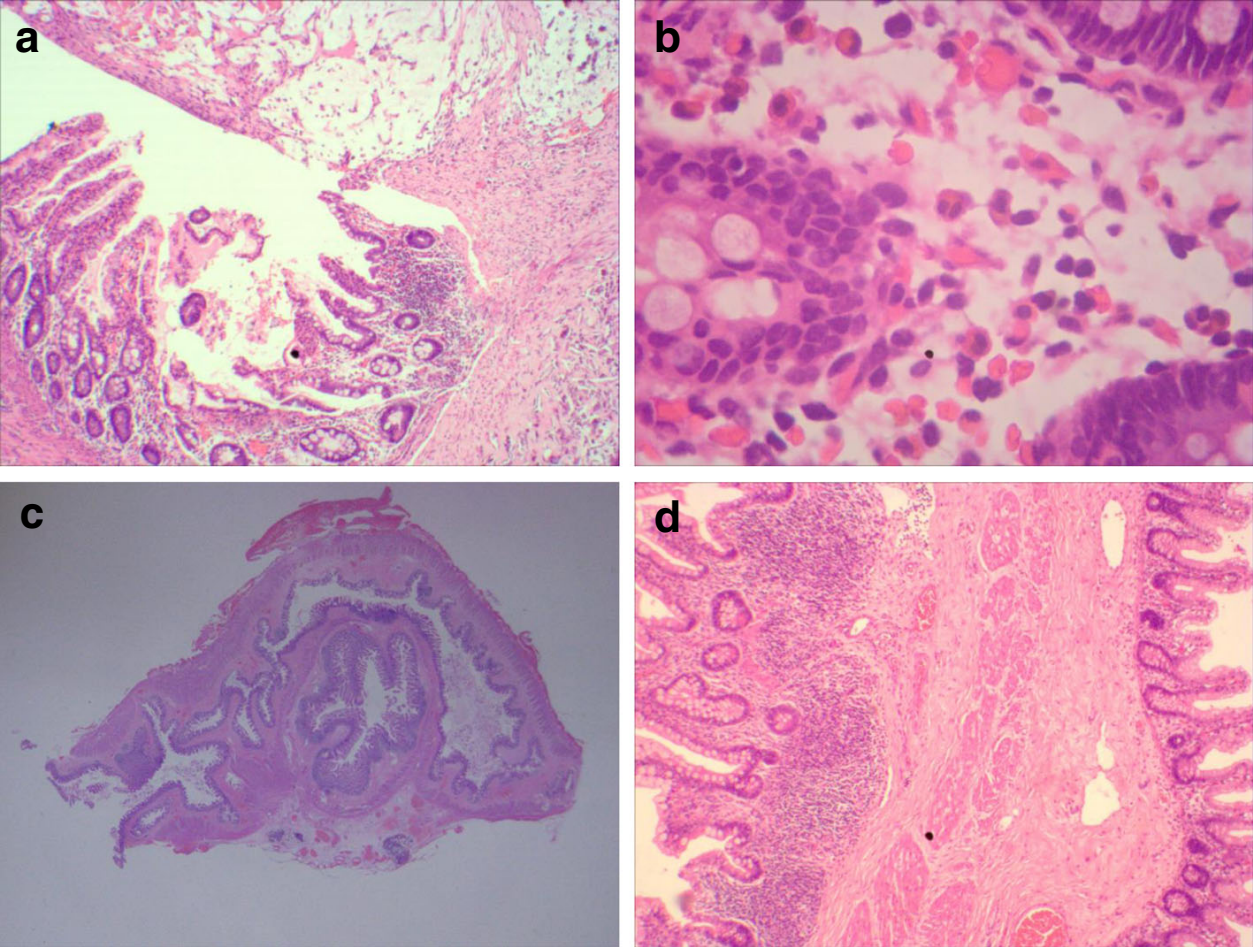
Histopathologic examination revealed appendiceal perforation and caecal duplication for Case 1. **a** HE staining showed the absence of the appendiceal wall, incomplete swelling mucosa and fibrino-suppurative infiltrate in the lumen. (HE, × 40). **b** The microscopic section showed neutrephil and eosinophilia infiltration in the mucosa of the gangrenous appendix. (HE, × 400). **c**, **d** The microscopic section showed the whole structure of caecal duplication in the lumen of the caecum (HE, original size), and there was complete mucosa, muscularis and serosa layer as same to the normal caecum. (HE, × 40)

Histological sections from the appendix showed ulceration of the mucosa with increased lympho-mononuclear cells in the lamia propria, hyperemia and edema in the submucosa, and organizing serositis with perforation (Fig. 2a, b). Notably, cecal duplication was also observed in the lumen of the cecum (Fig. 2c, d). The patient recovered uneventfully and was discharged 4 weeks after the operation. During the following 2 years, he remained clinically well with normal development.

### Case 2

**Fig. 3:**
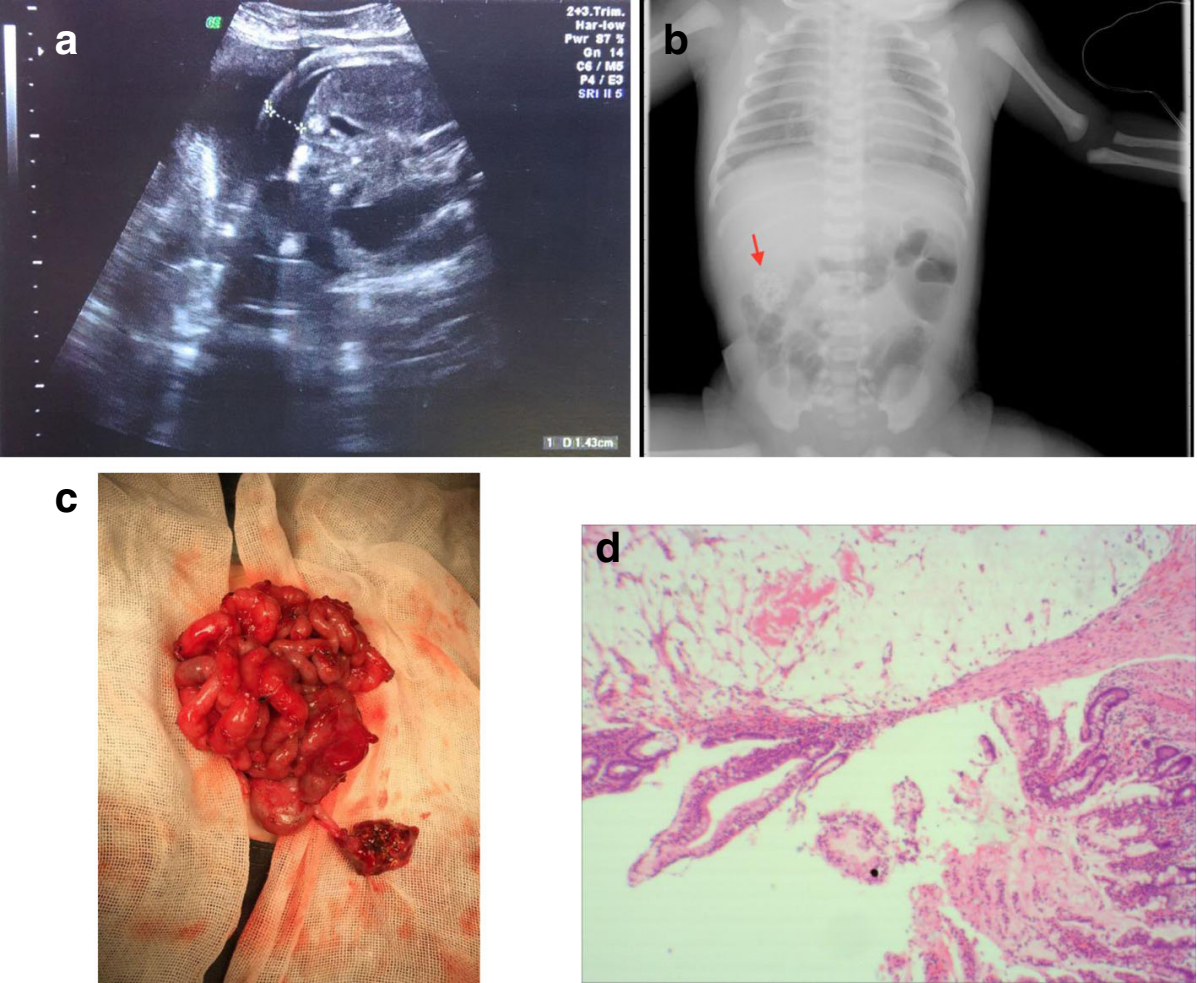
Imaging examination, intraoperative and Histopathological finding for Case 2. **a** Prenatal US showed the ascites in the abdominal cavity with 14.3 mm in depth. **b** After birth, the abdominal plain suggested a calcification foci in the right lower abdominal cavity (Red Arrow). **c**, **d** Intraoperative finding showed gangrenous appendicitis near the tip adhere to the calcification lesion and histopathological results revealed that perforated appendix with incomplete mucosa, tissue hyperemia and infiltration of inflammatory cells. (HE, × 40)

A 3560-g male newborn was smoothly delivered by cesarean section to a 32-year-old gravida 2, para 1. At mid-pregnancy (about 27 weeks of gestation), prenatal US examination showed fetal ascites with a maximum depth of 11 mm, and follow-up US examination indicated that the ascites had been gradually increasing (Fig. 3a). At the 37^th^ week of gestation, he was born by cesarean section.

After birth, physical examination of the neonate revealed a mildly distended abdomen. During abdominal palpation, a hard mass of about 4 × 3 × 3 cm was felt in the right middle abdomen. A plain abdominal radiograph suggested calcification foci in the abdominal cavity (Fig. 3b). The white blood cell count and CRP concentration were within normal limits. The patient was suspected to have MP, and abdominocentesis produced 150 ml of turbid ascites and confirmed the diagnosis. The neonate then underwent urgent laparotomy.

Similar to the intraoperative findings in Case 1, after complete suction of the ascites, we found that the intestines were firmly stuck together, especially in the right lower abdomen. We released the adhesions and carefully cleaned up the calcified lesions, then found a gangrenous appendix with perforation near the tip. Intraoperative exploration revealed no other structural anomalies (Fig. 3c). A standard appendectomy with abdominal drainage was then performed. Histopathological examination revealed a perforated appendix with incomplete mucosa, tissue hyperemia, and infiltration of inflammatory cells (Fig. 3d). The patient recovered well after 3 weeks of treatment, and his development remained normal during 10 months of follow-up.

## Discussion

Only a few cases of prenatal appendicular perforation have been reported since Martin first described this condition in 1986 (Table 1) [3–6]. The incidence is extremely low in fetuses, mainly because of the morphology of the appendix (funnel-shaped with a wider lumen at the junction of the cecum), which is also considered to reduce the morbidity of appendicitis in the neonatal period [7]. Preoperative diagnosis of this lesion is very difficult because it has no specific clinical presentation. Several acute abdominal symptoms in postpartum neonates, including vomiting, abdominal distention, and fever, were common among the previously reported cases [3–6]. Because of the progress that had been made in antenatal care measurements, we had suspected meconium ileus in utero in our two patients because of continuous intestinal dilation and ascites formation. Both of our patients underwent abdominocentesis after birth, allowing for a basic diagnosis of MP. Clinicians should be aware of the abnormal presentation of the fetus and maintain constant attention and follow-up when a congenital digestive tract malformation or illness during pregnancy is suspected.

**Table 1:** Reported cases of fetal appendiceal perforation

First Author/ Reference	Age/Gender	Symptoms/Signs	Treatment	Pathological found	Combined Disease	Follow-up/period
L.W. Martin [3]	21 days/F	Vomiting, fever, Abdominal distention/ Mass, RLQ	Appendectomy	AP with a local calcified pseudocyst	–	Uneventful/1 year
K.L. Narasimharao [4]	12 days/F	Constipation, Abdominal distention/Mass, RLQ	Ileocaecal resection	AP with local calcification	–	Unkown
R.R. Lebel [5]	23 weeks+ 3 days (gestation)/F	Spontaneous fetal demise / Hydrops fetalis	–	AP with local hydrops	Parvovirus B-19 infected	–
Valentina Pastore [6]	3 days/F	Tachycardia, fever, vomiting, abdominal distension	Ladd’s procedure with AP	gangrenous AP	Patau’s syndrome	Died 2 months after surgery (*Klebsiella pneumoniae*)

Abbreviations in the table: Age/Gender: *F* Female, *M* Male, Symptoms/Signs: *RLQ* right lower quadrant of the abdomen, *AP* appendiceal perforation

The pathophysiology of a perforated appendicitis in fetus or neonates may differ from that in older children. According to the literature, it may occur secondary to obstructive lesions such as colonic/anal atresia, Hirschsprung’s disease, or ischemic lesions such as necrotizing enterocolitis (NEC) [6, 8, 9]. Additionally, some reports have stated that it may be associated with aplasia of the muscularis mucosa, primary vascular insufficiency, or infective diseases (cytomegalovirus and chorioamnionitis) [5, 10, 11]. In our first patient, the postoperative pathological findings suggested an integrated structure of cecal duplication in the cecum. To the best of our knowledge, this is the first report of fetal appendicitis with enteric duplication. It reasonably explains the cause of obstruction of the distal cecum.

Once a patient has clear surgical indications, such as signs of peritonitis, abnormal diagnostic abdominocentesis, or imaging/laboratory examination findings that indicate a severe situation, laparotomy should be performed as soon as possible because MP has a high mortality rate, and early management is needed [12, 13]. In our first patient, after dissecting extensive adhesions and removing calcifications, ileocecal resection was preferred because the terminal ileum was perforated and the local tissue showed a severe inflammatory reaction. In the postoperative follow-up, we did not find that the baby had apparent watery stools, and we considered that this finding was due to the adequate length of the residual colon.

## Conclusion

We have herein presented two rare cases of MP caused by fetal appendiceal perforation. Preoperative diagnosis of appendiceal perforation in utero is very difficult; however, clinicians should be aware of any abnormal gastrointestinal symptoms in the fetus and maintain constant attention. For patients with severe MP, an early operation with careful intraoperative exploration is necessary.

## Abbreviations

CRP: C-reactive protein
MP: Meconium peritonitis
MRI: Magnetic Resonance Imaging
NEC: Necrotizing enterocolitis
US: Ultrasound
